# Number of conditioning trials, but not stimulus intensity, influences operant conditioning of brain responses after total knee arthroplasty

**DOI:** 10.1002/ksa.12480

**Published:** 2024-09-26

**Authors:** Kazandra M. Rodriguez, Chandramouli Krishnan, Riann M. Palmieri‐Smith

**Affiliations:** ^1^ School of Kinesiology University of Michigan Ann Arbor Michigan USA; ^2^ Department of Physical Medicine and Rehabilitation Michigan Medicine Ann Arbor Michigan USA; ^3^ Biomedical Engineering University of Michigan Ann Arbor Michigan USA; ^4^ Michigan Robotics Institute University of Michigan Ann Arbor Michigan USA; ^5^ Department of Mechanical Engineering University of Michigan Ann Arbor Michigan USA; ^6^ Department of Physical Therapy College of Health Sciences, University of Michigan-Flint Flint Michigan USA; ^7^ Department of Orthopaedic Surgery Michigan Medicine Ann Arbor Michigan USA

**Keywords:** cortical excitability, knee replacement, motor learning, neurorehabilitation, psychomotor performance

## Abstract

**Purpose:**

The primary purpose of this randomized, cross‐sectional study was to determine whether operant conditioning of motor evoked torque (MEP_TORQUE_) in individuals with total knee arthroplasty (TKA) increases quadriceps MEP_TORQUE_ responses within a single session and induces acute corticospinal adaptations by producing sustained increases in MEP_TORQUE_ after training. A secondary purpose was to determine if these changes were affected by the stimulus intensity and number of training trials.

**Methods:**

Thirty participants were block‐randomized into one of three groups based on the participant's active motor threshold (100%, 120%, and 140%) to evaluate the effect of stimulus intensity. Participants received three blocks of conditioning trials (COND), where they trained to increase their MEP_TORQUE_. Control (CTRL) transcranial magnetic stimulation pulses were provided before and after each COND block to establish baseline corticospinal excitability and to evaluate the effect of the number of training trials. Two MEP_TORQUE_ recruitment curves were collected to evaluate the effect of up‐conditioning on acute corticospinal adaptations.

**Results:**

TKA participants were able to successfully increase their MEP_TORQUE_ in a single session (*F*_3,81_ = 10.719, *p* < 0.001) and induce acute corticospinal adaptations (*F*_1,27_ = 20.029, *p* < 0.001), indicating sustained increases in quadriceps corticospinal excitability due to operant conditioning. While the stimulus intensity used during training did not affect the ability to increase MEP_TORQUE_ (*F*_2,26_ = 0.021, n.s.) or its associated acute adaptations (*F*_2,27_ = 0.935, n.s.), the number of training trials significantly influenced these outcomes (*F*_3,81_ = 10.719, *p* < 0.001; *F*_3,81_ = 4.379, *p* = 0.007, respectively).

**Conclusion:**

Operant conditioning is a feasible approach for improving quadriceps corticospinal excitability following TKA. While any of the three stimulus intensities evaluated in this study may be used in future operant conditioning interventions, using a low or moderate stimulus intensity and 150 training trials are recommended to improve treatment efficiency and patient adherence.

**Level of Evidence:**

Level II.

**Abbreviations**
ACL: anterior cruciate ligament
AMT: active motor threshold
AUC: area under the curve
COND: conditioning trials
CTRL: control trials
Hz: hertz
kg: kilograms
m: metres
MEP: motor evoked potential
MEP_TORQUE_
: motor evoked torque
ms: milliseconds
MVIC: maximum voluntary isometric contraction
NMES: neuromuscular electrical stimulation
N‐m: Newton‐metres
POST: following operant conditioning
PRE: prior to operant conditioning
RC: recruitment curve
SD: standard deviation
TKA: total knee arthroplasty
TMS: transcranial magnetic stimulation
%: percentage

## INTRODUCTION

Quadriceps dysfunction is common after total knee arthroplasty (TKA) and is associated with poor knee function [3, 5, 18]. Reduced corticospinal excitability could decrease the neural drive to the muscle, thereby contributing to this dysfunction [20]. Therefore, interventions that facilitate corticospinal excitability could potentially restore quadriceps strength and voluntary activation after TKA. While neuromuscular electrical stimulation (NMES) is commonly used for improving quadriceps function [16], it does not directly target the corticospinal pathway. Techniques like transcranial electrical stimulation and high‐frequency repetitive transcranial magnetic stimulation (r‐TMS) can directly increase corticospinal excitability [32] but are clinically impractical because of inconsistent effects [7], high costs, or safety concerns (e.g., seizures) [23]. Thus, identifying new, safe, and effective ways to enhance corticospinal excitability is crucial for improving quadriceps function after TKA.

Operant conditioning of motor evoked potentials (MEPs) is an emerging technique that has shown the potential to enhance muscle strength, activation, and motor function by safely and directly modulating corticospinal excitability [2, 15, 25, 26, 31]. Therefore, studying the feasibility of this approach and its application after TKA could be particularly beneficial in the rehabilitation of individuals with TKA. Moreover, identifying the optimal dosage parameters, such as the stimulus intensity and the number of training trials required for an effective intervention, could pave the way for clinical translation. Therefore, the primary purpose of this study was to determine if a single session of operant up‐conditioning of the corticospinal pathways in individuals with TKA can increase corticospinal excitability and induce acute neural adaptations in the corticospinal pathways. It was hypothesized that individuals with TKA would successfully increase (i.e., up‐condition) their motor evoked torque (MEP_TORQUE_) responses within a single session, which would be paralleled by acute neural adaptations (i.e., aftereffects). A secondary purpose of this study was to evaluate if the stimulus intensity and number of training trials used during operant up‐conditioning influenced these outcomes. Based on previous work in individuals with anterior cruciate ligament (ACL) reconstruction [21], it was hypothesized that the up‐conditioning ability and the associated aftereffects would be influenced by the number of training trials but not by stimulus intensity. Specifically, we hypothesized that the up‐conditioning ability and the associated aftereffects would be highest in the final conditioning block and lowest in the first conditioning block, regardless of the stimulus intensity.

## MATERIALS AND METHODS

### Participants

A total of 30 individuals with TKA participated in this study following screening (Supporting Information S1: Figure S1). Participant demographic characteristics, including self‐reported race and ethnicity, are reported in Tables 1 and 2. This sample size was determined a priori using the group means and standard deviations (Supporting Information S2: Table S1) for quadriceps MEP_TORQUE_ derived from previously published data evaluating the effect of block and stimulus intensity on improvements in MEP_TORQUE_ during operant conditioning in ACL reconstructed individuals [21]. Based on this data, a power analysis in General Linear Mixed Model Power and Sample Size (GLIMMPSE 3.0) software [13] indicated that a total sample size of *N* = 27 (9 per group) provided a power (1‐β) > 90% to detect a significant main effect for conditioning block. The following assumptions were made for this analysis: (1) mean and standard deviation values were equal to those observed in the data for ACL reconstruction, (2) repeated measure correlation coefficients were equal to those observed in the data for ACL reconstruction (Supporting Information S3: Table S2), (3) homogenous variances and covariances, and (4) an adjusted *p*‐value of 0.0167 to account for three post hoc simple effects comparisons (one at each of the three conditioning blocks) for the main effect of group.

**Table 1:** Demographic characteristics of participants with TKA.

Variable	TKA (*n* = 30) Mean ± SD
Age (years)	60.4 ± 4.6
Sex (females/males)	18/12
Race, *n* (%)	
Black or African American	2 (6.7)
White	28 (93.3)
Ethnicity, *n* (%)	
Hispanic or Latino/a	2 (6.7)
Not Hispanic or Latino/a	28 (93.3)
BMI (kg/m^2^)	31.2 ± 4.7
Tested leg (left/right)	18/12
Footedness (left/right)	3/27
Time since surgery (years)	4.1 ± 2.2

Abbreviations: BMI, body mass index; *n* = sample size, SD, standard deviation; TKA, total knee arthroplasty.

**Table 2:** Demographic characteristics of participants by group.

	100% AMT	120% AMT	140% AMT
	Mean ± SD	Mean ± SD	Mean ± SD
Group sample size, *n*	12	12	12
Age (years)	59.66 ± 6.39	61.83 ± 3.88	59.63 ± 2.80
Sex (females/males)	7/3	6/4	5/5
BMI (kg/m^2^)	29.99 ± 5.06	32.51 ± 3.12	31.09 ± 5.71
Time since surgery (years)	3.25 ± 1.93	4.28 ± 2.97	4.87 ± 2.38
Mass‐normalized quadriceps strength (N‐m/kg)	1.49 ± 0.40	1.33 ± 0.34	1.70 ± 0.41

Abbreviations: AMT, active motor threshold; BMI, body mass index; kg, kilogram; m, metres; *n*, sample size; N‐m, Newton‐metres; SD, standard deviation.

Participants were included if they were (1) aged 45–70 years and (2) underwent total knee replacement at least 12 months prior to the testing. Participants were excluded if they had (1) a cardiac pacemaker; (2) metal implants in the skull; (3) other recent lower‐extremity injury or lower‐extremity fracture; (4) body mass index ≥ 40 kg/m^2^; (5) a history of uncontrolled diabetes or hypertension; and/or (6) a history of unexplained recurrent headaches, seizures, recent head injury, medical or heart condition that could influence study outcomes or significant adverse reaction to TMS. Participants reviewed and signed a written informed consent document approved by the University of Michigan Institutional Review Board (HUM00166442) prior to enrollment. The procedures used in this study adhere to the tenets of the Declaration of Helsinki.

### Study overview

**Figure 1:**
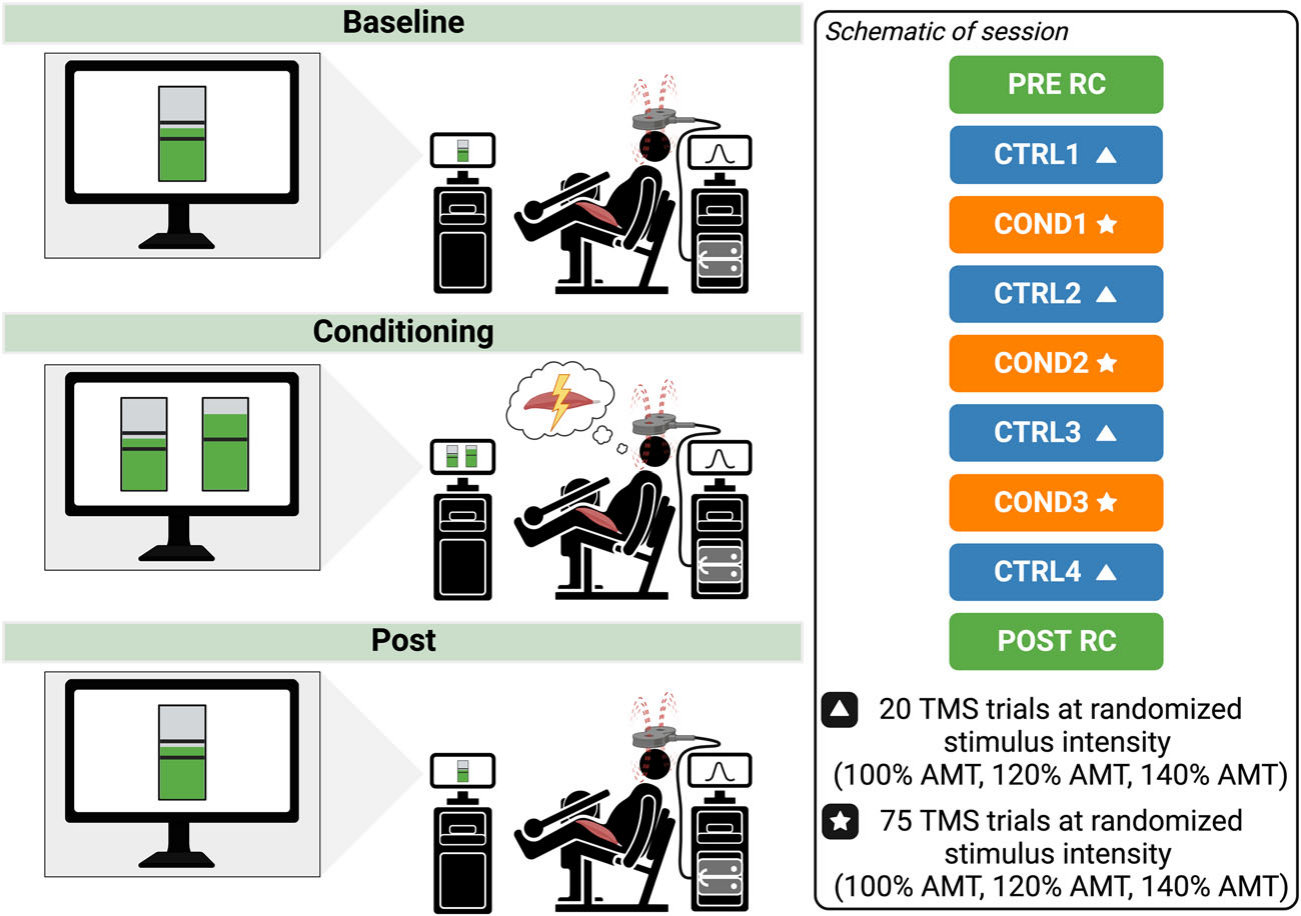
A schematic of the experimental protocol. AMT, active motor threshold; COND1, conditioning block 1; COND2, conditioning block 2; COND3, conditioning block 3; CTRL1, control block 1; MVIC, maximum voluntary isometric contraction; POST RC, recruitment curve collected at the end of the session; PRE RC, recruitment curve collected at the beginning of the session; TMS, transcranial magnetic stimulation.

A schematic of the study overview is illustrated in Figure 1.

This is a prospective cross‐sectional study with a randomized design (level II) designed to test the feasibility of a single session of operant up‐conditioning of the quadriceps motor evoked torque response to increase the motor evoked torque in individuals with TKA. Testing was performed in a research laboratory setting from March 2023 to October 2023. Participants were randomized to one of three groups based on the TMS intensity (% active motor threshold [AMT]) used during training: (1) 100% AMT; (2) 120% AMT; or (3) 140% AMT. The AMT was established as the minimum stimulus intensity needed to induce a motor evoked torque response in ≥50% of attempted trials (≥10 trials). All procedures were performed on the surgical leg. All study procedures for testing and training were identical between groups, except that the stimulus intensity used during training was manipulated across groups.

### Experimental protocol

During the operant conditioning intervention, participants were trained to increase the MEP_TORQUE_ of the quadriceps muscle on the surgical leg during a single session. Participants were seated and secured into an isokinetic dynamometer (Humac Norm, CSMi) with the trunk and knee set to 85 and 60 degrees of flexion, respectively. While secured into the dynamometer, a 110‐mm diameter double‐cone coil connected to a Magstim 200^2^ stimulator (Magstim Co Ltd) was used to elicit motor evoked torque (MEP_TORQUE_) responses. The coil was positioned over the primary motor cortex on the hemisphere contralateral to the tested leg and oriented to induce a posterior‐to‐anterior current flow in the cortex. The point located 2.0 cm posterior and 2.0 cm lateral to the vertex of the skull was determined as the temporary quadriceps hotspot location and marked on a fabric cap [14]. The coil was systematically moved from this temporary location to identify the hotspot (i.e., the location over the skull that resulted in the largest and most consistent motor evoked torque response during a small background contraction [12 N‐m for females, 16 N‐m for males]). Visual feedback was provided to participants to maintain a consistent background contraction (Supporting Information S1: Figure S2). The hotspot location was marked on the fabric cap to ensure consistent coil placement during the session. The AMT was established as the minimum stimulus intensity needed to induce a motor evoked torque response in ≥50% of attempted trials (≥10 trials) using the relative‐frequency method [8]. The AMT was used to establish the stimulus intensity during the control (CTRL) and conditioning (COND) blocks.

Once the hotspot location and AMT were established, a baseline TMS recruitment curve (PRE) was performed and recorded at eight different intensities (70%–140% AMT). Immediately after, a baseline control block of 20 TMS trials was collected as has been done previously [15, 24]. The baseline control block (CTRL1) was used to determine the participant's baseline corticospinal excitability and to calculate the initial criterion value (i.e., the 50th percentile value of the motor evoked torque responses from the 20 control trials) for the subsequent conditioning block. During the control block, participants were instructed to focus on sustaining a consistent background contraction as previously described and received a TMS pulse to the contralateral hemisphere at the assigned stimulus intensity (100% AMT, 120% AMT or 140% AMT) when the contraction was sustained. No feedback regarding performance was provided to participants during the control blocks.

Following the baseline control block, a block of 75 conditioning trials was collected. The conditioning block was similar to the control block, except the participant was instructed to use motor imagery to try and train the corticospinal pathways to increase the motor evoked torque responses above the criterion value. Examples were given, such as the quadriceps feeling a ‘burn’ or imagining the contraction of the quadriceps when doing exercises (e.g., leg presses, squatting, etc.) or performing an exercise action (e.g., walking up a hill or cycling with high resistance). Motor imagery visualizations utilized by participants are represented in Supporting Information S1: Figure S3. The initial criterion value for the first conditioning training block (COND1) was established as the 50th percentile value of the participant's MEP_TORQUE_ during the control block. The participant's performance during the preceding block was used to dynamically establish the criterion value for the subsequent training blocks. The criterion value was established such that if MEP_TORQUE_ amplitudes during the current block were similar to the MEP_TORQUE_ amplitudes during the preceding training block, ~50% of the trials would be successful (i.e., above the criterion value) [15, 28]. Participants received visual feedback regarding their performance on each trial, indicating whether participants were successful at increasing the motor evoked torque responses above the criterion value. The feedback bar increased and turned green if successful or decreased and turned red if unsuccessful (Supporting Information S1: Figure S2). Participants also received dynamic feedback on the percentage of successful trials during the current conditioning block. The participant's goal during each conditioning block was to obtain a trial success rate ≥60% and earn a small monetary incentive to achieve this target (20 cents for each trial greater than 60%). Researchers also provided verbal encouragement and positive verbal feedback during conditioning blocks.

A total of three conditioning blocks were performed (COND1, COND2 and COND3) with the previously described procedures. Following each conditioning block, a control block was also collected to evaluate the effect of number of conditioning trials (CTRL2, CTRL3 and CTRL4). After the final control block, a second TMS recruitment curve (POST) was performed to determine the acute adaptations in corticospinal excitability due to the operant conditioning intervention.

### Data management

Custom‐written LabVIEW (National Instruments Corp.) programs were used to perform all data collection and analysis. Torque data and TMS synchronization pulses were sampled at 1000 Hz. Torque signals were low‐pass filtered (10 Hz, 4th order) using a zero‐lag digital Butterworth filter [6]. Torque data were segmented from 200 ms prior to the stimulation over a window of 500 ms for each of the stimulations. For each block, ensemble averages of the segmented torque data were used to construct an average torque curve. The magnitude of the MEP_TORQUE_ amplitude was computed as the peak twitch torque after accounting (i.e., subtracting) for the background contraction torque. The PRE and POST TMS recruitment curves from 100% AMT to 140% AMT were also used to determine the area under the curve (AUC) of the MEP_TORQUE_. Raw quadriceps MEP_TORQUE_ and the AUC data from the authors' laboratory demonstrate good‐to‐excellent (intraclass correlation coefficient [ICC] = 0.660–0.947) and excellent test–retest reliability (ICC = 0.923), respectively, in individuals with knee surgeries [22]. In addition, trial‐to‐trial variability for baseline corticospinal excitability (i.e., MEP_TORQUE_ during CTRL1) demonstrates similar variability (coefficient of variation = 26.5%) to previous work [4, 22].

### Statistical analysis

Descriptive statistics were used to evaluate the distribution and variation of the outcome variable (i.e., within‐session change in MEP_TORQUE_). Graphical methods such as histograms, residual plots, and Q–Q plots were used to visually inspect the data and to confirm assumptions of constant variance and normality. The assumption of normality for the outcome variable was also confirmed using the Shapiro–Wilks test. There was no missing data for any of the outcome variables. A linear mixed model with block (CTRL1, COND1, COND2 and COND3), group (100%, 120%, and 140% AMT), and block × group as fixed effects and subject as a random effect was used to determine if TKA individuals were able to successfully improve the MEP_TORQUE_ in a single session and if stimulus intensity and number of training trials affected the improvements in MEP_TORQUE_. The MEP_TORQUE_ during the baseline control (CTRL1) and the conditioning blocks were used as the dependent variable, and the MEP_TORQUE_ during the baseline control block (CTRL1) was used as a covariate in the model. A second linear mixed model with time (PRE, POST), group (100%, 120%, and 140% AMT), and time × group as fixed effects and subject as a random effect was used to determine if operant up‐conditioning elicited significant increases in acute corticospinal excitability and whether stimulus intensity affected the corticospinal adaptations during a single session. For this model, the AUC of the MEP_TORQUE_ was used as the dependent variable. Finally, a separate linear mixed model with block (CTRL1, CTRL2, CTRL3, and CTRL4), group (100%, 120%, and 140% AMT), and block × group as fixed effects and subject as a random effect was used to determine the effect of number of conditioning trials and stimulus intensity on the change in MEP_TORQUE_ during the session. The MEP_TORQUE_ during the control blocks was used as the dependent variable, and the MEP_TORQUE_ during the baseline control block (CTRL1) was used as a covariate in the model. A significance level of alpha = 0.05 was used for all analyses. Planned post hoc tests using a Šidák correction were used for pairwise comparisons when a significant main or interaction effect was observed. For the effect of the block on the up‐conditioning ability and acute neural adaptations, pairwise comparisons were only made with respect to the baseline control (CTRL1) block, as the purpose of this study was to establish the minimum number of training trials required to increase the MEP_TORQUE_ and induce acute neural adaptations.

## RESULTS

### Changes in MEP_TORQUE_ during conditioning and the effect of stimulus intensity

**Figure 2:**
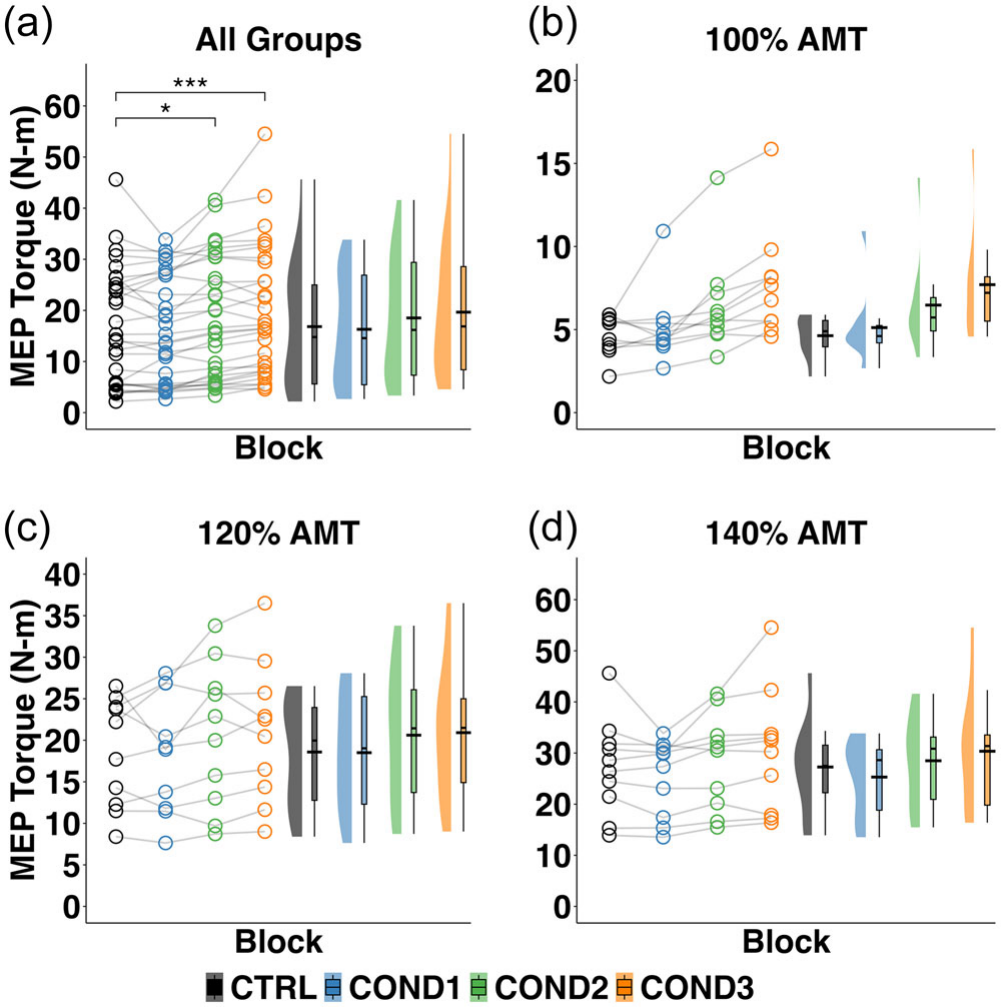
Raincloud plots depicting the distribution of the distribution of MEP_TORQUE_ during the baseline control block immediately before operant conditioning (CTRL1) and all three conditioning blocks (COND) for (a) all groups, (b) stimulus intensity group 100% AMT only, (c) stimulus intensity group 120% AMT only, and (d) stimulus intensity group 140% AMT only. Open circles represent data points from each individual participant. Black horizontal lines represent the mean across all participants for each timepoint. AMT, active motor threshold; COND1, conditioning block 1; COND2, conditioning block 2; COND3, conditioning block 3; CTRL1, control block 1; MEP_TORQUE_, motor evoked torque; N‐m, newton‐metres. *, Significant increase from CTRL to COND2, regardless of stimulus intensity (*p* < 0.05). ***, Significant increase from CTRL to COND3, regardless of stimulus intensity (*p* < 0.001).

Ensemble averaged data from a representative participant and group data on the ability to up‐condition the quadriceps MEP_TORQUE_ is shown in Supporting Information S1: Figure S4. During conditioning, a significant main effect of the block (*F*_3,81_ = 10.719, *p* < 0.001) on the MEP_TORQUE_ amplitude was detected (Figure 2). Post hoc analysis demonstrated that MEP_TORQUE_ amplitude during COND2 and COND3 were significantly higher than CTRL1 (*t*_81_ = 2.109, *p* = 0.038; *t*_81_ = 3.448, *p* ≤ 0.001, respectively, CTRL1^1^: mean [standard error]): 16.826 [0.627], COND2^1^: 18.524 [0.627], COND3^1^: 19.656 [0.627]) while COND1 was not (COND1^1^: 16.307 [0.627], *t*_81_ = 0.224, *p* = n.s.), indicating that the participants were able to successfully up‐condition their corticospinal excitability and that the number of training trials affected this ability. Specifically, participants were able to increase their MEP_TORQUE_ following 150 and 225 training trials, suggesting at least 150 training trials are needed to improve quadriceps corticospinal excitability. During conditioning, no significant effect of group (i.e., stimulus intensity) (*F*_2,26_ = 0.021, n.s.) or the interaction between block and group (*F*_6,81_ = 0.710, n.s.) was detected (Figure 2).

### Acute changes in corticospinal excitability and the effect of stimulus intensity and number of training trials

**Figure 3:**
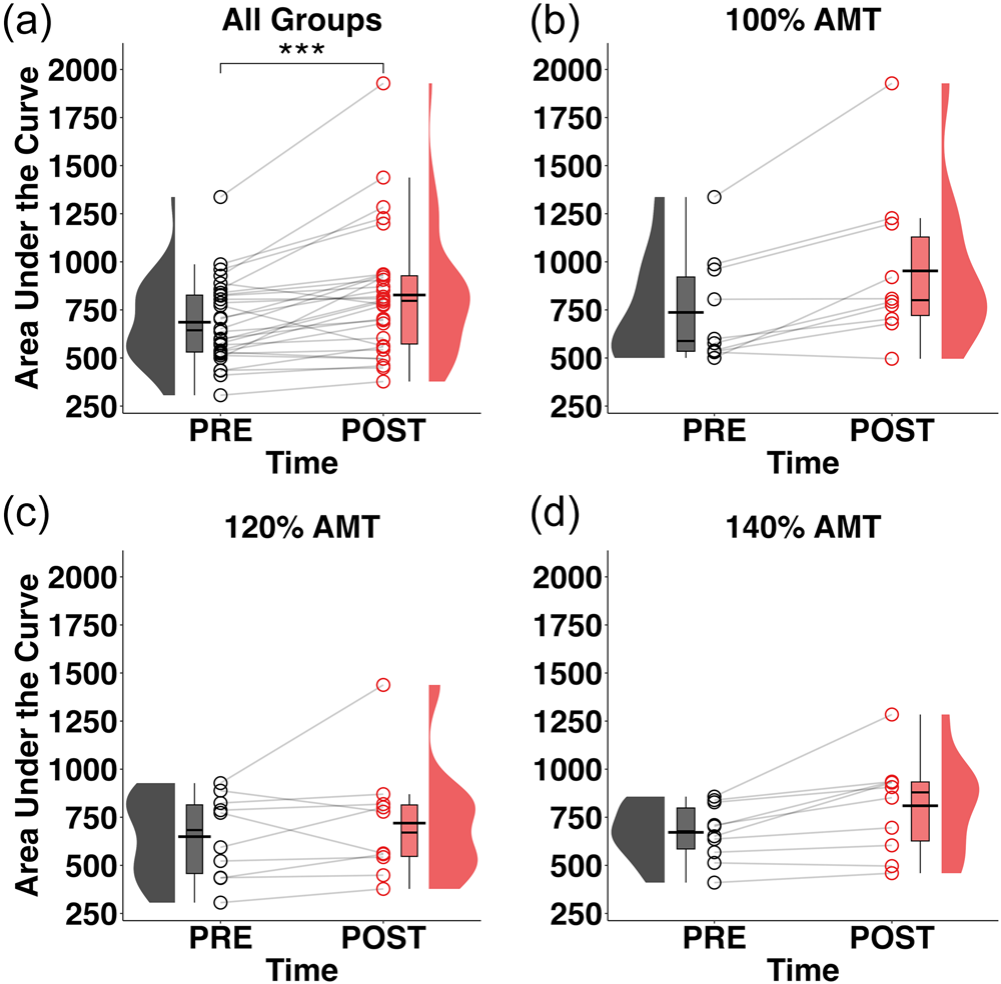
Raincloud plots depicting the distribution of the area under the curve of MEP_TORQUE_ prior to up‐conditioning procedures (PRE) and immediately after up‐conditioning procedures (POST) for (a) all groups, (b) stimulus intensity group 100% AMT only, (c) stimulus intensity group 120% AMT only, and (d) stimulus intensity group 140% AMT only. Open circles represent data points from each individual participant. Black horizontal lines represent the mean across all participants for each timepoint. AMT, active motor threshold; MEP_TORQUE_, motor evoked torque; N‐m, newton‐metres; POST, following operant conditioning; PRE, prior to operant conditioning. ***, Significant increase from PRE to POST (*p* < 0.001).

A significant main effect of time (*F*_1,27_ = 20.029, *p* < 0.001) on the MEP_TORQUE_ AUC was detected (Figure 3). Post hoc analysis revealed that MEP_TORQUE_ AUC increased following the intervention compared to prior to the intervention (*t*_27_ = 3.73; *p* < 0.001; PRE^1^: 685.888 [51.076], POST^1^: 827.387 [51.076]), indicating that operant up‐conditioning of MEP_TORQUE_ resulted in acute corticospinal adaptions in individuals with TKA (Figure 3). However, no significant effect of group (i.e., stimulus intensity) (*F*_2,27_ = 0.935, n.s.) or the interaction between group and time (*F*_2,27_ = 1.769, n.s.) was detected (Figure 3).

**Figure 4:**
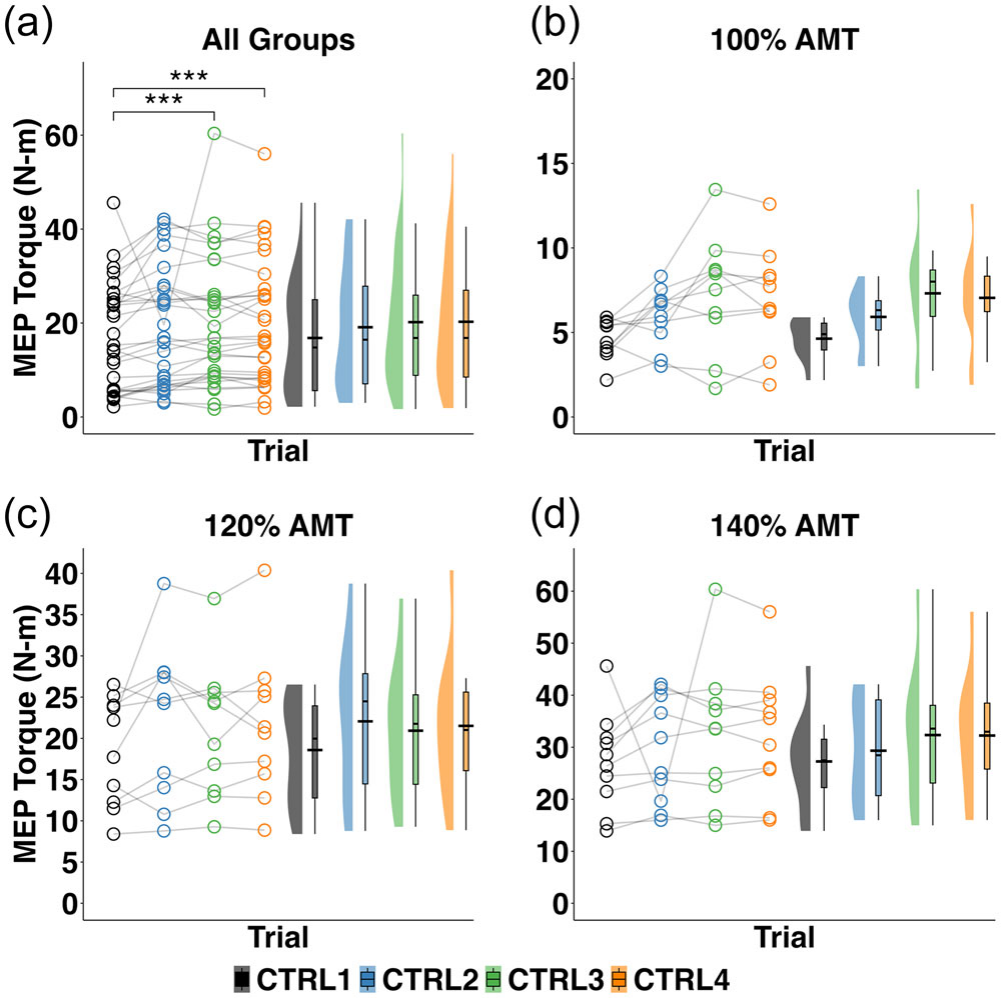
Raincloud plots depicting the distribution of MEP_TORQUE_ during the baseline control block immediately before operant conditioning (CTRL1) and after each conditioning block (CTRL2, CTRL3 and CTRL4) for (a) all groups, (b) stimulus intensity group 100% AMT only, (c) stimulus intensity group 120% AMT only, and (d) stimulus intensity group 140% AMT only. Open circles represent data points from each individual participant. Black horizontal lines represent the mean across all participants for each timepoint. AMT, active motor threshold; CTRL1, control block 1; CTRL1, control block 1; CTRL2, control block 2; CTRL3, control block 3; CTRL4, control block 4; MEP_TORQUE_, motor evoked torque; N‐m, newton‐metres. ***, Significant increase from CTRL1 to CTRL4, regardless of stimulus intensity (*p* < 0.001).

During the control blocks, a significant main effect of the block (*F*_3,81_ = 4.379, *p* = 0.007) on the MEP_TORQUE_ amplitude was detected (Figure 4). Post hoc analysis revealed that MEP_TORQUE_ amplitude during CTRL3 and CTRL4 were significantly higher than CTRL1 (*t*_81_ = 2.719; *p* = 0.008; *t*_81_ = 2.822, *p* = 0.006, respectively, CTRL1^1^: 16.826 [0.865], CTRL3^1^: 20.194 [0.865], CTRL4^1^: 20.270 [0.865]) while CTRL2 was not (*t*_81_ = 1.607, *p* = 0.112, CTRL2^1^: 19.104 [0.865]), indicating that the acute neural adaptations due to operant conditioning were dependent on the number of training trials used in the training. Specifically, acute neural adaptions were observed following 150 and 225 training trials, suggesting at least 150 training trials are needed to improve quadriceps corticospinal excitability. In addition, no significant effect of group (i.e., stimulus intensity) (*F*_2,26_ = 0.084, *p* = 0.920) or the interaction between block and group (*F*_6,81_ = 0.607, n.s.) was detected (Figure 4).

## DISCUSSION

The most important finding of the current study was that individuals with TKA were able to increase their quadriceps corticospinal excitability within a single session of operant up‐conditioning, which was paralleled by acute neural adaptations of the corticospinal pathway. These changes were influenced by the number of training trials, but not by the stimulus intensity used during training. Collectively, these findings suggest that operant conditioning of the quadriceps MEP_TORQUE_ is a feasible approach to improve corticospinal excitability in individuals with TKA, regardless of the stimulus intensity used. However, at least 150 training trials are needed to induce acute neural adaptations.

An important finding of this study was that quadriceps MEP_TORQUE_ increased during the conditioning blocks compared to the control block. This finding indicates that individuals with TKA can successfully increase their corticospinal responses and induce acute neural adaptations (i.e., aftereffects) within a single session of operant conditioning. This finding is consistent with recent evidence reporting individuals with ACL reconstruction were able to successfully increase their MEP_TORQUE_ responses within a single session of operant conditioning, which were paralleled by sustained increases in corticospinal excitability (i.e., aftereffects) after training [21]. This finding is particularly encouraging, considering that individuals with TKA are typically older than other patient populations with quadriceps dysfunction (e.g., ACL injury or surgery, meniscus injury, etc.) and that older adults typically have difficulty in learning cognitively demanding tasks such as operant conditioning of motor evoked responses. A growing body of evidence demonstrates that operant conditioning interventions have the potential to improve muscle strength and gait biomechanics [15, 26, 27], which are known to be affected following TKA [1, 17]. Notably, these alterations in quadriceps strength and gait biomechanics may persist well after surgery [17, 30], suggesting that the current standard of care is inadequate to fully restore motor function after surgery. Therefore, long‐term operant conditioning interventions that target the motor evoked torque may be a promising supplement to rehabilitation following TKA.

When evaluating whether the ability to up‐condition the corticospinal pathways is dependent on the stimulus intensity used during training, this study found that TMS stimulus intensity did not influence the up‐conditioning ability (i.e., the ability to increase the quadriceps MEP_TORQUE_ during training) in a single session or the acute neural adaptations. This finding is consistent with a previous study, which found that stimulus intensity had no effect on the ability to up‐condition the quadriceps MEP_TORQUE_ or the acute neural adaptations in individuals with ACL reconstruction [21]. Together, these findings suggest that other factors are more likely to influence the ability to up‐condition the quadriceps MEP_TORQUE_ and the associated acute neural adaptations. Future investigation of the factors that influence a participant's ability to up‐condition would provide valuable insight for developing effective operant conditioning protocols. Nevertheless, this study establishes that operant conditioning protocols for individuals with TKA may effectively use any of the three stimulus intensities studied, although 120% AMT appears to be a good compromise between variability (i.e., signal‐to‐noise) and participant discomfort.

When evaluating whether the up‐conditioning ability and acute neural adaptations were influenced by the number of training trials used during the session, the current study found that the up‐conditioning ability was dependent on the number of training trials. Specifically, increases in MEP_TORQUE_ were observed only after 150 training trials. The acute neural adaptations were also observed following 150 conditioning trials, but there were no further increases for the remainder of the session. The diminishing returns for the up‐conditioning ability and acute neural adaptations suggest there may be a ‘ceiling effect’, after which additional conditioning trials may not elicit further increases in corticospinal excitability or acute adaptations within a single session. In addition, it is plausible that other parameters for the number of conditioning trials that were not studied herein (e.g., 100 or 200 trials) may induce even greater acute neural adaptations than those observed in the current study. Regardless, the clinical implication of this finding suggests that 150 and 225 conditioning trials both appear to be sufficient to elicit acute neural adaptations in individuals with TKA. However, using 150 conditioning trials would increase the feasibility of operant conditioning in a clinical setting. In order to develop an operant conditioning intervention with sustained therapeutic benefits, long‐term investigations are critically needed to establish whether 150 conditioning trials are sufficient to induce the long‐term neural adaptations that appear to precede improvements in motor function [15, 26].

There are some potential limitations to the current study that warrant consideration. First, we examined the influence of stimulus intensity on the ability to increase the quadriceps MEP_TORQUE_ during a single session. Although this study found that stimulus intensity does not appear to influence the ability to up‐condition or its aftereffects, it is possible that cumulative effects due to multiple training sessions could reveal stimulus intensity as an influential factor in the ability to up‐condition. The authors also cannot comment on whether stimulus intensities that were not tested in the current study (e.g., >140% AMT) may influence the ability to improve the MEP_TORQUE_ after TKA, as we only tested three intensities (100%, 120%, and 140% of AMT). In addition, musculoskeletal imaging was not collected in these participants due to funding constraints. Thus, we cannot determine what role pre‐operative and post‐operative muscle quality and size [11, 12, 29], knee alignment phenotype [9, 10, 19], and other potentially relevant measures may have on the current study findings. Further, muscle fatigue may have influenced the increase in quadriceps MEP_TORQUE_ observed during the session. However, this is unlikely, given previous work in individuals with knee surgery indicates that mild fatigue does not result in an increase in MEP_TORQUE_. Hence, the increases in corticospinal excitability during the session were more likely due to the motor imagery practice during operant conditioning.

While the operant conditioning intervention evaluated in the current study is not yet fully translatable to a clinical setting, the results have meaningful clinical implications. This study is the first to establish the feasibility of operant conditioning after TKA, indicating that operant conditioning may be a valuable supplement to standard rehabilitation for addressing quadriceps weakness and atherogenic muscle inhibition (i.e., voluntary activation deficits) after TKA. Moreover, this study establishes the practical dosage that is needed for consistent and sustained increases in corticospinal excitability. Specifically, the findings indicate that the efficiency of this intervention could be improved by reducing the conventional dosage of 225 trials to 150 trials, which may be more practicable in a clinical setting. Given that operant conditioning has been shown to improve muscle strength and gait [26], this intervention has the potential to improve surgical and functional outcomes after TKA. For example, higher quadriceps strength is associated with faster walking speeds, improved stair climbing ability, and lower Timed Up and Go times [5]. Hence, future work is critically needed to explore long‐term operant conditioning interventions as a means to improve quadriceps function in individuals with TKA in order to provide strong clinical evidence to support its use in a rehabilitation setting.

## CONCLUSION

In summary, the current study found that individuals with TKA were able to successfully up‐condition the quadriceps MEP_TORQUE_ within a single operant conditioning training session, which was paralleled by acute neural adaptations in the corticospinal pathway. These findings reveal that operant conditioning of the MEP_TORQUE_ may be a feasible approach for improving corticospinal excitability after TKA. While any of the three stimulus intensities tested could be used, using a low or moderate stimulus intensity (i.e., 100%–120% AMT) and 150 training trials are recommended to improve treatment efficiency and patient adherence to the intervention.

## AUTHOR CONTRIBUTIONS

K.M.R., C.K., R.P.S. conceived and designed research; K.M.R. performed experiments; K.M.R., C.K., R.P.S. analyzed data; K.M.R., C.K., R.P.S. interpreted results of experiments; K.M.R., C.K., R.P.S. prepared figures; K.M.R. drafted manuscript; K.M.R., C.K., R.P.S. edited and revised manuscript; K.M.R., C.K., R.P.S. read and approved final version of manuscript.

## CONFLICT OF INTEREST STATEMENT

The authors declare no conflict of interest.

## ETHICS STATEMENT

This study conforms to the guidelines of the Declaration of Helsinki. Participants reviewed and signed a written informed consent document approved by the University of Michigan Institutional Review Board (HUM0016642) prior to enrollment.

## Supporting information

Supporting information.

Supporting information.

Supporting information.

## Data Availability

Data are available upon request.

## Footnotes

1 Reported as mean ± pooled standard error of the mean.
