# Supplementary material for: Number of conditioning trials, but not stimulus intensity, influences operant conditioning of brain responses after total knee arthroplasty
Source: Knee Surg Sports Traumatol Arthrosc. 2024 Sep 26;33(3):967–76. doi: 10.1002/ksa.12480 (PMC11848966; doi:10.1002/ksa.12480)

Supplementary Fig. 1 Consolidated Standards of Reporting Trials (CONSORT) flow diagram.

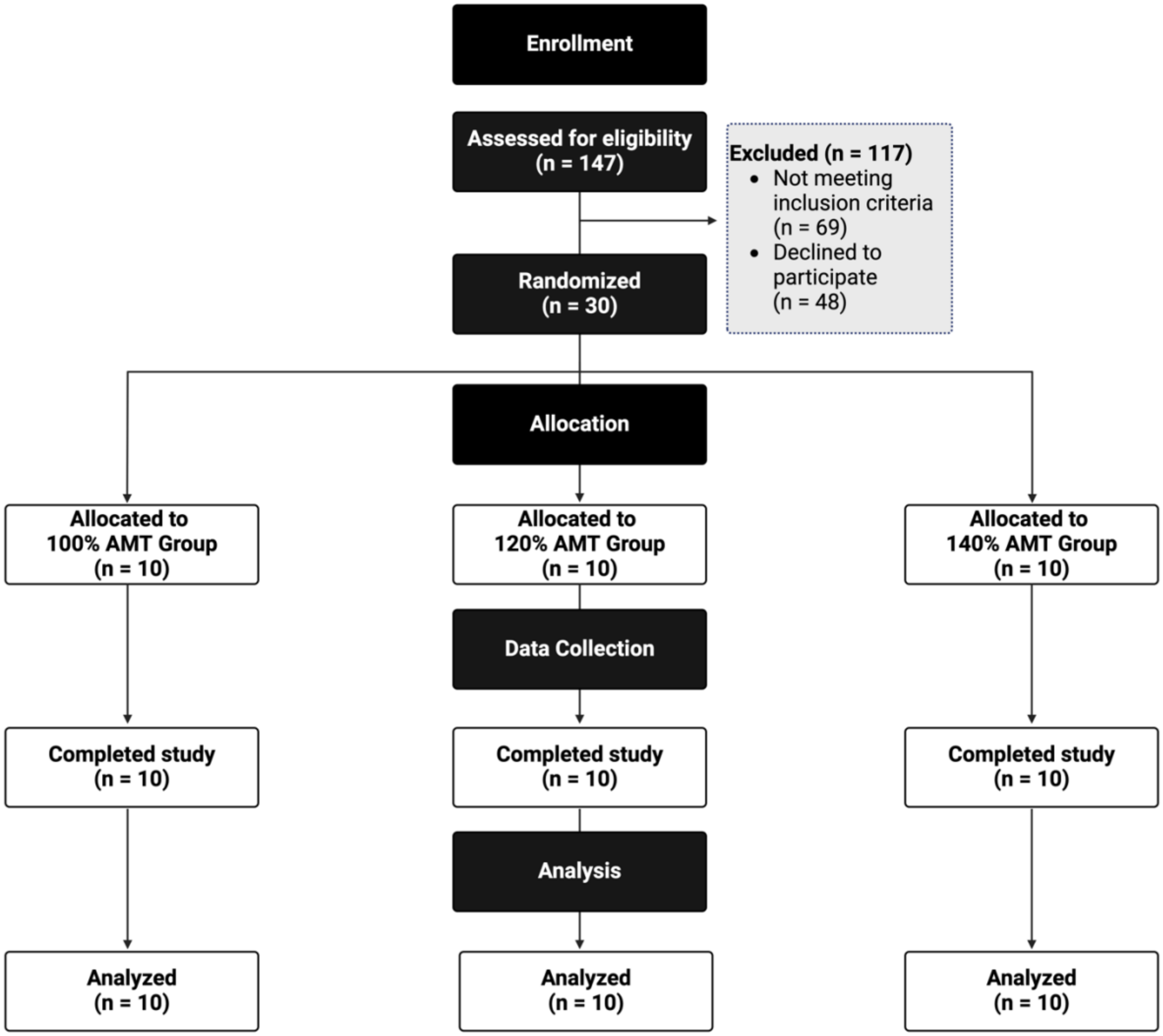

Supplementary Fig. 2 On the left is a schematic indicating visual feedback for a small background contraction (12 N-m for females, 16 N-m for males), which was shown for both the control and conditioning blocks. The participant's torque output is indicated by the green bar, which must stay within the force target range. Below the force target, participants can see the number of completed trials. On the right is a schematic illustrating visual feedback of a successful or unsuccessful trial provided to participants during the conditioning blocks. The conditioning bar will turn green when the most recent training trial successfully increased the MEP_{TORQUE} (i.e., MEP_{TORQUE} greater than the conditioning target). The conditioning bar will turn red when the most recent training trial was unsuccessful in increasing the MEP_{TORQUE}. Below the feedback bar, participants can see their current success rate, which updates after each conditioning trial and resets at the start of each conditioning block. *Abbreviations*: MEP_{TORQUE}, motor evoked torque; N-m, Newton-meters.

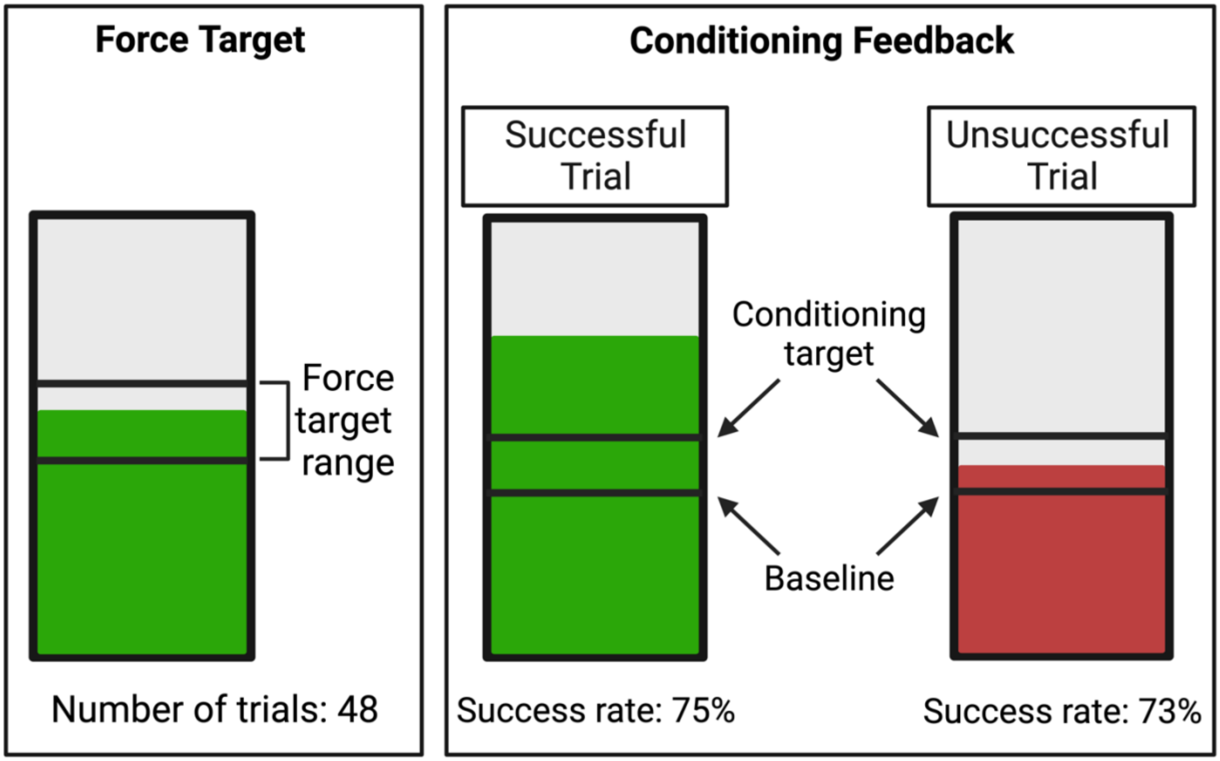

Supplementary Fig. 3 Wordcloud depicting the motor imagery visualizations used by participants during up-conditioning procedures. Words with a larger font size correspond to visualizations that were more frequently used by participants, while words with a smaller font size were less frequently used.

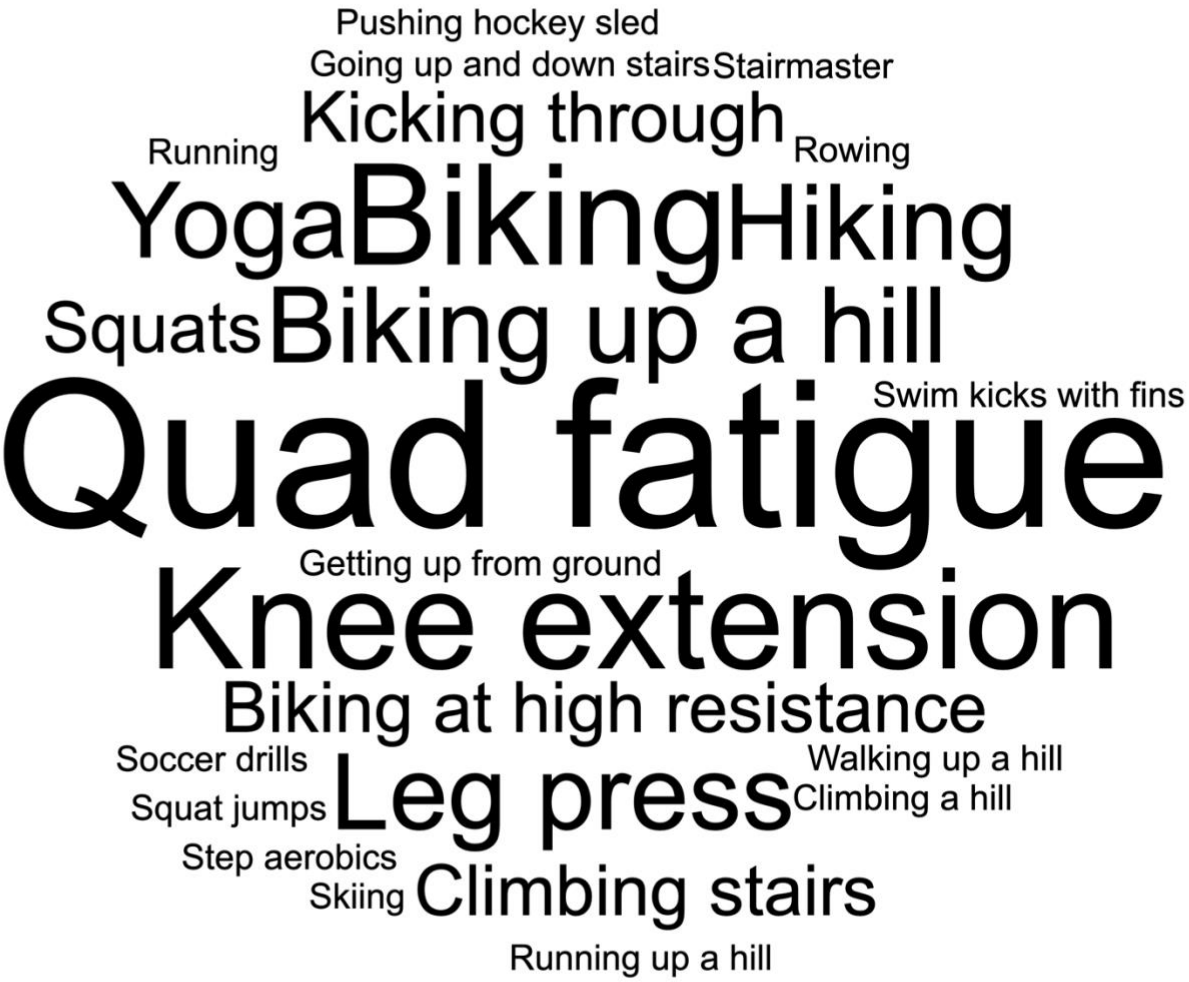

Supplementary Fig. 4 Ensemble averaged motor evoked torque (MEP_{TORQUE}) plots for a single subject **(a-c)** and for all participants **(d-f)**. Data from a representative participant for **a)** ensemble averaged MEP_{TORQUE} for the baseline control block (CTRL1) and all three conditioning blocks (COND); **b)** ensemble averaged MEP_{TORQUE} for all four control blocks (CTRL); **c)** MEP_{TORQUE} recruitment curves prior to operant conditioning (PRE) and following operant conditioning (POST). Ensemble averaged group data are shown in panels **d, e and f**. *Abbreviations:* AMT, active motor threshold; COND1, conditioning block 1; COND2, conditioning block 2; COND3, conditioning block 3; CTRL1, baseline control block 1; CTRL2, control block 2; CTRL3, control block 3; CTRL4, MEP_{TORQUE}, motor evoked torque; N-m, newton-meters; ms, milliseconds; PRE, prior to operant conditioning; POST, following operant conditioning; TMS, transcranial magnetic stimulation

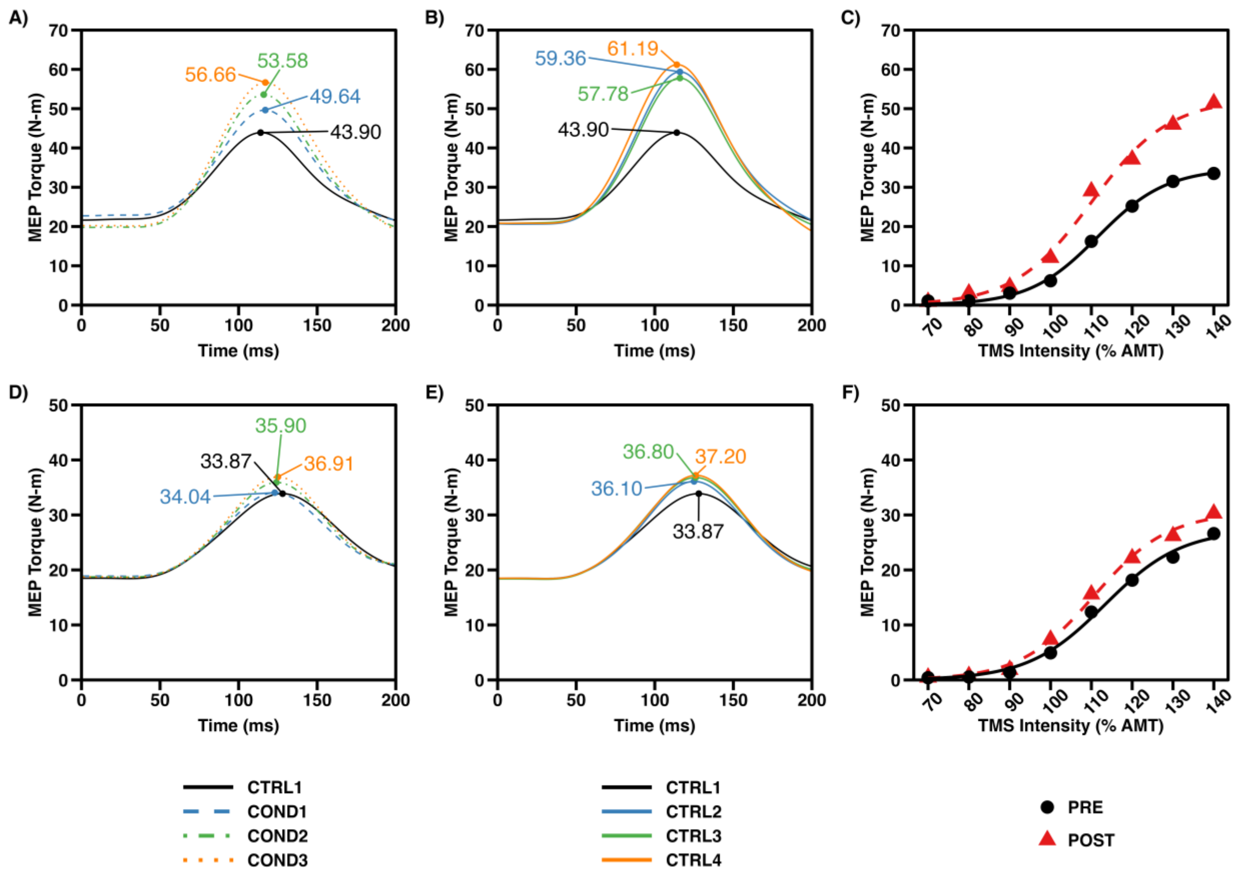

Supplement: Supplementary file 1 — Supporting information. [file KSA-33-967-s003.pdf]
