# Supplementary material for: Number of conditioning trials, but not stimulus intensity, influences operant conditioning of brain responses after total knee arthroplasty
Source: Knee Surg Sports Traumatol Arthrosc. 2024 Sep 26;33(3):967–76. doi: 10.1002/ksa.12480 (PMC11848966; doi:10.1002/ksa.12480)
Supplement: Supplementary file 2 — Supporting information. [file KSA-33-967-s001.pdf]

Supplementary Table 1 Group means for quadriceps MEP_{TORQUE} derived from unpublished data evaluating the effect of block and stimulus intensity on improvements in MEP_{TORQUE} during operant conditioning in ACL reconstructed individuals. A standard deviation $\sigma = 10.4$ for the outcome variable (quadriceps MEP_{TORQUE}) was derived from the unpublished data and used for the variability across outcomes. In addition, a standard ratio = 1 was assumed for all blocks.

**Group**	**Block 1**	**Block 2**	**Block 3**	**Block 4**
	Mean	Mean	Mean	Mean
100 % AMT	3.9	4.3	5.3	5.5
120 % AMT	21.0	25.4	28.1	27.4
140 % AMT	26.9	30.6	32.9	32.8

Abbreviations: ACL, anterior cruciate ligament; AMT, active motor threshold; MEP_{TORQUE}, motor evoked torque.
