# Supplementary material for: Number of conditioning trials, but not stimulus intensity, influences operant conditioning of brain responses after total knee arthroplasty
Source: Knee Surg Sports Traumatol Arthrosc. 2024 Sep 26;33(3):967–76. doi: 10.1002/ksa.12480 (PMC11848966; doi:10.1002/ksa.12480)
Supplement: Supplementary file 3 — Supporting information. [file KSA-33-967-s002.pdf]

Supplementary Table 2 The unstructured correlation matrix for block derived from unpublished data evaluating the effect of block and stimulus intensity on improvements in MEP_{TORQUE} during operant conditioning in ACL reconstructed individuals.

Block 1	Block 2	Block 3	Block 4
1	0.92	0.86	0.86
0.92	1	0.97	0.96
0.86	0.97	1	0.99
0.86	0.96	0.99	1

Abbreviations: ACL, anterior cruciate ligament; MEP_{TORQUE}, motor evoked torque.
